# The effect of an automated patient engagement solutions (APES) on step-by-step parental communication around time of discharge and length of stay

**DOI:** 10.1038/s41372-026-02633-w

**Published:** 2026-03-30

**Authors:** Beatrice M. Stefanescu, Amanda B. Deford, Lakeisha J. Boyd, Angela E. Cromlich, Susan E. Henderson-Sears, Heather J. Bruckman, Samantha R. O’Neill

**Affiliations:** 1 Department of Pediatrics, Indiana University School of Medicine and Riley Hospital for Children at Indiana University Health, Indianapolis, IN USA; 2 Department of Biostatistics and Health Data Science, Indiana University School of Medicine and Richard M. Fairbanks School of Public Health, Indianapolis, IN USA; 3 Informatics and Information Systems Department, Indiana University Health, Indianapolis, IN USA

**Keywords:** Health services, Health care

## Abstract

**Objectives:**

To determine the impact of an automated patient engagement solutions (APES) pathway at discharge on parental communication and length of stay (LOS) compared to traditional approach.

**Study design:**

This quasi-experimental study took place in a level 4 neonatal intensive care unit. The APES at discharge (APES-D) pathway was implemented on January 18, 2022. Infants admitted in year prior formed the Pre-Intervention group. Post-implementation infants were categorized as APES-D Users (opted in) or Non-users (opted out).

**Results:**

Pre-Intervention and APES-D groups had similar LOS (*p* = 0.54). APES-D Users had shorter LOS than APES-D Non-users (*p* = 0.007) and Pre-Intervention and APES-D Non-users combined (*p* = 0.03). Time between discharge document completion and actual discharge was longer in APES-D (*p* < 0.001). Commercial insurance was associated with shorter LOS (*p* < 0.001). Among APES-D Users, 91.7% engaged with the platform.

**Conclusion:**

APES-D was associated with high parental engagement and shorter LOS, but usage varied by social determinants, warranting further study.

## Introduction

The transition from the Neonatal Intensive Care Unit (NICU) to home represents a critical phase in the care journey for both premature and critically ill infants and their families [1]. This period can be overwhelming as parents navigate complex medical care, discharge instructions [2], and an emotionally charged environment [3]. Effective preparation during this phase is essential for the infant’s continued health and the well-being of the family, yet studies show parents often struggle with understanding and managing discharge tasks, leading to heightened stress, confusion [4, 5], and increased hospital readmission risk [6]. Parents must complete numerous preparatory tasks within a limited timeframe to ensure a safe and successful transition home, making communication key [7, 8]. As the date of discharge approaches, they receive substantial educational information from physicians, advanced practice providers, nurses, case managers, and other healthcare professionals via face-to-face meetings, written materials, phone calls, and various other forms of communication. While these efforts are designed to ensure the infant’s safety, disorganized or inconsistent communication can cause discharge delays, adverse outcomes, or hospital readmissions if instructions are not properly followed [9]. The reliance on paper-based discharge processes further complicates matters, especially for families managing infants with complex medical needs.

In recent years, the shift toward digital communication has become increasingly evident [10], particularly among younger, digitally native parents [11]. Traditional paper-based instructions often fail to engage this generation, who prefer quick, efficient interactions via email, text messages, or social media [12]. Consequently, healthcare engagement strategies must evolve to align with these preferences, leveraging technology to foster better connections and improve the support necessary for successful home transition [13]. While digital patient engagement platforms have shown promise in other areas of healthcare, their use in the NICU remains limited [14, 15]. However, these automated platforms have the potential to streamline communication [16], provide continuous access to care instructions [17], deliver timely reminders, and offer real-time guidance, ultimately reducing parents’ cognitive load and enhancing their preparedness for post-discharge care [18].

This study examines the potential impact of an automated platform on improving parents’ ability to manage NICU discharge tasks. By evaluating the efficacy of this digital tool, we aim to provide insight into how such platforms can contribute to smoother transitions from the NICU to home care and impact length of stay (LOS), ultimately promoting better health outcomes for neonates and enhancing the family’s experience. The main objectives of the study were to evaluate the effects of a step-by-step automated messaging pathway on parental communication around time of discharge to home and LOS compared to traditional approach, by analyzing infants admitted the year before and after the Automated Patient Engagement Solutions at Discharge (APES-D) implementation. Secondary objectives included examining differences between APES-D users and APES-D non-users in LOS and specific time-related discharge tasks during the intervention period.

## Methods

### Study design, setting, and participants

This is a cohort study with a pre-/post-intervention study groups design. The study took place in an urban academic center level IV NICU in the USA. The NICU is a 60-acute bed unit within a free-standing children’s hospital with approximately 620 admissions every year. The study followed the recommended STROBE guidelines [19]. The “Pre-Intervention” group included eligible infants admitted in the year prior to APES-D implementation on January 18, 2022. Post-intervention or APES-D group included infants admitted in the year following tool implementation. The APES-D group was further divided into “APES-D Nonuser” group (comprising infants of parents who opted out APES -D), and “APES-D User” group (comprising infants of parents who used the APES-D). Parents who did not own a mobile phone or other computer-based technology to connect to APES could not opt-in therefore were excluded from the study. Parents in whom English or Spanish were not their primary language were still offered APES, as many spoke one of these languages as a secondary language.

### Traditional discharge approach

In our unit, we encourage starting the parental discharge education process as soon as clinically appropriate after admission. However, discharge planning often begins closer to discharge due to factors such as caregiver availability, patient severity of illness, and caregiver stress. Standard discharge education included in-person viewing the purple period of crying and CPR skills videos, receiving neonatal care instructions, and learning about car seat safety and safe sleep practices. Additional education varies based on the infant’s medical needs and may include but is not limited to nasogastric or gastrostomy-tube care, formula preparation, and medication administration. Often, a significant amount of information was delivered in a short time, typically within 24 h of discharge, with caregiver availability, stress, and information retention influencing the process. Education is reinforced using the teach back method and demonstration check offs to assess caregiver understanding.

Discharge specialists serve as a dedicated resource for bedside staff and families, offering expert assistance throughout the discharge process. Their specialized knowledge in planning and procedures helps coordinate tasks, address potential delays, and assist with documentation, medication education, and follow-up care – ensuring more optimal support at this transition point.

### APES development and implementation team

The APES Development and Implementation Team consisted of a project manager, a clinical informatics specialist with Twistle build certification, a neonatologist, a discharge planning nurse specialist, an advance practice provider, a parent support representative, a case manager, and a project coordinator. The team met at least monthly for the eight months preceding APES implementation.

The APES-D used Twistle (Twistle by Health Catalyst ™, South Jordan, Utah) as the secure automated messaging platform to send timed messages, educational documents, and videos to parents of infants preparing for discharge to home. These documents were saved in the patient’s Twistle account for as long as parents needed them, and they could be reviewed at any time. The Twistle account is accessible using a web browser or downloadable mobile app. The automated platform was HIPAA compliant and integrated seamlessly with the Electronic Medical Records (EMR). Its initial message was deployed by an EMR order. The same documents in paper format and videos used with the traditional approach were sent via the automated pathway.

Our automated patient solutions platform uses dashboards to visualize and monitor operational efficiency and key performance indicators. The discharge specialists are tasked with tracking each infant’s discharge task progression on the dashboard, detecting potential issues early, and intervening proactively with the bedside nurse when delays arise. Bedside education including teach-back and demonstration checkoffs continued to be used to assess caregiver understanding of educational points.

### Study measures

The primary outcome was the LOS and the time to discharge task completion. Secondary prespecified outcomes include the comparison of the effect of the social determinants of health (including ethnicity and race, sex, primary language, and insurance type) on LOS. Data on cardiac [20], genetic or structural congenital anomalies [21], and surgical diagnoses [22] were collected, as these factors can contribute to prolonged length of stays. For the intervention period, the same outcomes were measured when comparing the APES-D U and APES-D Non-U groups. Patient demographic and baseline characteristics, as well as discharge task type and completed discharge task times were obtained from the EMR.

### Statistical analysis

Descriptive statistics were generated to report infant socio-demographics and outcome measures. Frequency and percentage values were given for categorical measures while continuous variables were measured using mean, median, standard deviation, range, and inter-quartile range values. General linear models with random effects to account for patients with multiple admissions were fit to compare hospital length of stay pre and post intervention and across APES-D user group, race, sex, ethnicity, language, and insurance. The remaining outcome variables were compared pre and post intervention and across APES-D group, race, sex, ethnicity, language, and insurance groups at first admission using Mann–Whitney U and Kruskal-Wallis tests. Sub analyses of the effect of APES-D User group on the time between discharge order and completion of the discharge documents at first admission utilized Mann–Whitney U tests and multivariable linear regression to examine the results after excluding documents completed before the discharge order, excluding documents completed after the discharge order, and with length of stay as a covariate. When applicable, non-parametric tests or log transformations were used for skewed continuous variables. A 5% significance level was used for all analyses. All statistical analyses were performed using SAS version 9.4 (SAS Institute, Inc., Cary, NC) and RStudio version 4.2.3 (RStudio, Boston, MA).

## Results

**Fig. 1:**
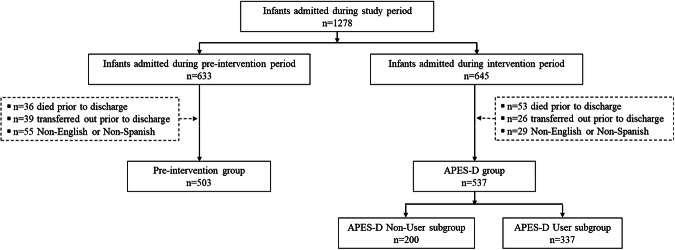
Study flowchart showing enrollment of infants; APES-D, automated patient engagement solutions.

Flowchart depicting infant enrollment process is shown in Fig. 1. A total of 1040 infants were included in the study period, with 503 infants in the pre-intervention period and 537 infants in the intervention period. There was no significant difference between study groups with respect to gestational age, birth weight, sex, ethnicity, or race (Table 1). No significant differences were observed between study groups regarding cardiac, genetic/congenital anomalies, or surgical diagnoses. There were more infants of English-speaking parents (*p* = 0.01) and on Public or Combined Public/Private insurance (*p* < 0.001) in the Pre-Intervention group compared to the APES-D group. Time between completion of discharge tasks and discharge was shorter in the pre-intervention period (*p* < 0.001).

**Table 1:** Patient baseline and sociodemographic characteristics, and selected discharge time-completion tasks at first admission among the Pre-Intervention and APES-D study groups.

Patient characteristic	Pre-Intervention group (*N* = 503)	APES-D group (*N* = 537)	*p* value
Gestational Age (weeks)	37 (34, 39)	37 (35, 39)	0.25
Birth Weight (grams)	2830 (1980, 3412.50)	2901 (2116.50, 3374.25)	0.73
Female sex	210 (41.75%)	236 (43.95%)	0.47
Ethnicity			0.18
Hispanic or Latino	55 (11.00%)	74 (13.78%)	
Not Hispanic or Latino	433 (86.60%)	456 (84.92%)	
Unknown/Refused	12 (2.40%)	7 (1.30%)	
Race			0.24
Black or African American	88 (17.50%)	88 (16.39%)	
White	368 (73.16%)	392 (73.00%)	
Other/Multiracial	16 (3.18%)	30 (5.59%)	
Unknown/Refused	31 (6.16%)	27 (5.03%)	
Language			0.01
English	478 (95.03%)	494 (91.99%)	
Spanish	20 (3.98%)	21 (3.91%)	
Other	4 (0.80%)	21 (3.91%)	
Cardiac diagnosis	159 (31.61%)	167 (31.10%)	0.86
Genetic/congenital anomaly diagnosis	71 (14.12%)	63 (11.73%)	0.25
Surgical diagnosis	251 (49.90%)	251 (46.74%)	0.31
Insurance Status			< 0.001
Combined Public/Private	36 (7.16%)	5 (0.93%)	
Private	131 (26.04%)	183 (34.08%)	
Public	336 (66.80%)	349 (64.99%)	
Time between completion of last discharge task and discharge (days)	0.26 (0.10, 1.18)	0.90 (0.12, 2.13)	<0.001

All continuous values are presented as median (IQR) and categorical values as number (%).

The median LOS was shorter in the APES-D group versus the Pre-Intervention group (11.7 days versus 15.9 days) and not significantly different (*p* = 0.54). Although 37.2% (200) of infants in the APES-D group did not use the automated platform. When APES-D Non-User and Pre-Intervention groups were combined, LOS was significantly shorter in APES-D Users (15.1 days versus 19.53 days; *p* = 0.03).

The APES-D group was a combination of APES utilizers (APES-D User) and non-utilizers (APES Non-Users) subgroups (Table 2). Infants in the APES-D User subgroup had higher birth weights (2980 g versus 2736 g; *p* = 0.024) and a higher proportion of parents with English as their primary language (95.6% versus 86%; *p* < 0.001) than those in the APES-D Non-User subgroup (Table 2). While a lower proportion of infants in the APES-D User subgroup had a cardiac diagnosis (27.41% versus 38.05%, *p* = 0.01), the frequency of genetic/congenital anomaly and surgical diagnoses was similar between the subgroups. Additionally, APES-D User subgroup had a significantly shorter LOS (20 days versus 30 days; *p* = 0.007). This difference remained statistically significant after adjusting for cardiac diagnosis (*p* = 0.012).

**Table 2:** Socio-demographics in the APES-D Nonuser and APES-D User subgroups during the intervention period.

Patient characteristic	APES-D Nonuser subgroup (*N* = 200)	APES-D User subgroup (*N *= 337)	*p* value
Gestational Age (weeks)	37 (34, 38)	37 (35, 39)	0.064
Birth Weight (grams)	2736 (1873.50, 3285)	2980 (2240, 3410)	0.02
Female sex	88 (44.00%)	148 (43.92%)	0.99
Ethnicity			0.88
Hispanic or Latino	26 (13.00%)	48 (14.24%)	
Not Hispanic or Latino	171 (85.50%)	285 (84.57%)	
Unknown/Refused	3 (1.50%)	4 (1.19%)	
Race			0.23
Black or African American	38 (19.00%)	50 (14.84%)	
White	138 (69.00%)	254 (75.37%)	
Other/Multiracial	15 (7.50%)	15 (4.45%)	
Unknown/Refused	9 (4.50%)	18 (5.34%)	
Language			< 0.001
English	172 (86.00%)	322 (95.55%)	
Spanish	7 (3.50%)	14 (4.15%)	
Other	21 (10.50%)	1 (0.30%)	
Cardiac diagnosis	78 (38.05%)	94 (27.41%)	0.01
Genetic/congenital anomaly diagnosis	21 (10.24%)	43 (12.54%)	0.41
Surgical diagnosis	105 (51.22%)	151 (44.02%)	0.11
Insurance Status			0.04
Combined Public/Private	3 (1.50%)	3 (0.89%)	
Private	57 (27.50%)	125 (37.09%)	
Public	140 (70.00%)	209 (62.02%)	
Time between Twistle discharge order and discharge (days)	NA	3.38 (1.31, 8.12)	
LOS (days)	33 (15, 82)	20 (10, 56)	0.007

All continuous values are presented as median (IQR) and categorical values as number (%).

There was no significant effect of ethnicity, race, sex, and language on LOS during the intervention period (Table 3). Infants with private insurance had the shortest LOS (14.12 days), followed public (18.42 days) and then combined public/private (92.58 days) (Table 3).

**Table 3:** Effect of sociodemographic factors on LOS during intervention period.

Predictor	Median (IQR)	*p* value
Overall	17.64 (7.56, 46.35)	
Parent Communication Method		0.003
APES-D Nonuser	22.72 (9.98, 58.17)	
APES-D User	15.08 (6.76, 36.73)	
Ethnicity		0.51
Hispanic or Latino	18.33 (9.03, 57.57)	
Not Hispanic or Latino	17.63 (7.53, 42.75)	
Race		0.082
Black or African American	22.29 (9.78, 75.05)	
White	16.60 (7.51, 44.69)	
Other/Multiracial	21.58 (10.83, 69.02)	
Sex		0.28
Female	17.65 (8.05, 54.18)	
Male	17.60 (7.32, 38.01)	
Language		0.34
English	17.28 (7.51, 44.21)	
Spanish	19.37 (10.03, 60.63)	
Other	23.74 (9.98, 111.13)	
Insurance at Discharge		0.038
Combined Public/Private	92.58 (34.22, 150.74)	
Private	14.12 (6.85, 35.89)	
Public	18.42 (7.99, 49.96)	

All values are presented as median (IQR) or number.

*APES-D* Automated Patient Engagement Solutions at Discharge, *LOS* length of stay.

In the intervention period, there was no significant effect of socio-demographic factors on time between date of discharge document completion or date discharge order placed in the EMR and the date of discharge from the hospital (Table 4).

**Table 4:** Effect of sociodemographic factors on time between date of discharge documentation completion and discharge order, and the date of discharge in the intervention subgroups.

Time between the date of discharge order and date of discharge documentation completion		
Predictor	Median (Q1, Q3)	*p* value
Overall	–0.77 (–1.96, –0.03)	
Parent Communication Method		0.49
APES-D Non-User	–0.30 (–2.19, –0.00)	
APES-D User	–0.82 (–1.93, –0.06)	
Ethnicity		0.30
Hispanic or Latino	–0.98 (–2.12, –0.06)	
Not Hispanic or Latino	–0.74 (–1.93, –0.03)	
Race		0.75
Black or African American	–0.31 (–2.21, –0.05)	
White	–0.84 (–2.04, –0.02)	
Other	–1.36 (–1.93, 0.00)	
Sex		0.94
Female	–0.35 (–2.12, –0.01)	
Male	–0.85 (–1.84, –0.04)	
Language		0.87
English	–0.74 (–1.95, –0.03)	
Spanish	–0.84 (–2.04, –0.18)	
Other	–1.36 (–1.93, 0.00)	
Insurance at Discharge		0.52
Combined Public/Private	–0.60 (–2.48, –0.14)	
Private	–0.87 (–2.03, –0.08)	
Public	–0.39 (–1.95, –0.01)	
**Time between date of discharge document completion and date discharge from the hospital**		
Predictor	Median (IQR)	*p* value
Overall	0.90 (0.12, 2.13)	
Parent Communication Method		0.83
APES-D Non-User	0.78 (0.09, 3.30)	
APES-D User	0.91 (0.15, 1.99)	
Ethnicity		0.29
Hispanic or Latino	1.09 (0.15, 2.28)	
Not Hispanic or Latino	0.88 (0.12, 2.05)	
Sex		0.99
Female	0.78 (0.11, 2.30)	
Male	0.96 (0.14, 1.97)	
Language		0.66
English	0.88 (0.12, 2.09)	
Spanish	1.05 (0.26, 2.17)	
Other	1.96 (0.08, 2.82)	
Race		0.96
Black or African American	0.37 (0.11, 2.28)	
White	0.96 (0.13, 2.20)	
Other/Multiracial	0.64 (0.21, 1.60)	
Insurance at Discharge		0.67
Combined Public/Private	0.66 (0.20, 2.55)	
Private	0.99 (0.17, 2.10)	
Public	0.78 (0.10, 2.15)	

All values are presented as median (IQR) or number.

*APES-D* Automated Patient Engagement Solutions at Discharge.

In a sub-analysis assessing whether LOS influenced the time between the individual discharge task order and the date of discharge document completion, LOS demonstrated a significant effect in both the bivariate model (*p* = 0.035), and the multivariable model (*p* = 0.038) controlling for APES-D User subgroup. In both models, the relationship was positive, indicating that as LOS increased, the time between discharge order and completion of documents also increased. The median time for all alerts, from the moment of message-received to message-read was 2.1 minutes.

## Discussion

This is the first study to examine the effect of a NICU automated engagement platform on discharge communication and LOS. We demonstrated its feasibility, with most parents promptly opening and reading the message alerts. Technologies using patient engagement platforms streamline communication [23], ensuring that families receive timely guidance and education [24], which can reduce complications and hospital readmissions [25, 26]. In line with this, Motamedi et al, in a systematic review of 12 studies evaluating the efficacy of computer-enabled discharge communication, found that these technological interventions were beneficial, leading to reduced medication errors and adverse events, as well as improved efficiency and enhanced satisfaction for both physicians and patients [27]. We hypothesized that improving family communication and support at discharge with these technologies could also help reduce LOS by providing early access to documents, making them accessible at any time, and ensuring parents are better prepared in advance. Our study is the first to show that an APES-D NICU not only improves communication but also shortens LOS in NICU infants.

Involving all key stakeholders, all bringing their own unique perspectives, in the co-design of patient-facing automated platforms is essential to ensure that these systems are user-friendly, effective and equitable [28]. Engaging stakeholders early in the design process helps align the platform with the real needs [29] and mitigates unintended biases or safety risks [30]. Our group had early multidisciplinary input and emphasized parental perspective, leading to more intuitive, patient-centered solutions that optimized engagement, improved outcomes, and we believe, overall healthcare experience for these parents. At least one former parent was present for all platform design meetings. Former NICU parents also served in an advisory capacity to review and provide feedback on the in-app communications to caregivers. Our automated platform’s dashboard allowed for monitoring the real-time progression of discharge tasks in APES-D users, which in turn allowed the discharge specialists to track task completion at-a-glance. When the progression appeared to slow down, the dashboard highlighted areas that needed attention. This enabled staff to prioritize certain tasks, reallocate resources, or engage the parents more to prevent delays and ensure timely discharge. Social determinants of health significantly influence technology usage, particularly in healthcare access and digital health adoption. Individuals with lower income may have limited access to smartphones, computers, or reliable internet, reflecting their ability to use telemedicine, patient portals, and health apps [31]. Individuals with lower health literacy may struggle with understanding and utilizing digital health tools, leading to disparities in healthcare engagement. It is not a requirement for the caregivers to download the app for our automated platform; just access to using a web browser is sufficient and helps reduce disparities. Despite what may be considered ease in access, 37.4% of eligible families opted out of APES-D. Although we did not collect data on specific reasons for opting out, previous studies conducted at our institution, which align with the broader literature, suggest that some of the reasons are related to social determinants of health [32, 33]. Addressing these disparities require policies and interventions that promote digital inclusion, such as improving internet access, providing digital literacy training, and designing user-friendly health technologies with key stakeholder input [34, 35].

Our study has several limitations. It had a quasi-experimental design, which may introduce a potential selection bias, and was conducted in a single academic health system, which may limit the generalizability of the findings to other settings with different populations, resources, or discharge processes. Additionally, only 62.6% of the families utilized the automated platform during the intervention period, limiting its overall reach and potential impact. Notably, a significant portion of families who were unable to opt-in were non-English, non-Spanish speakers. This highlights the need for tailored strategies to improve accessibility and usability for diverse patient populations with different linguistic and cultural backgrounds.

Evaluating readmissions and emergency department utilization would have provided further insights into the broader impact of the automated program; however, these outcomes could not be reliably assessed. As a statewide and regional referral center, many infants discharged from our NICU receive subsequent care outside our hospital system, and post-discharge utilization data from numerous external health systems were not accessible.

It was not an objective of the study to study the automated platform’s impact on parental cognitive ergonomics. Traditional discharge methods, which rely heavily on paper documents, can increase cognitive load and stress, as these materials are easily displaced and may not be available when parents need to review them. By contrast, an automated system like ours offers consistent access to essential information, potentially reducing mental strain and improving performance [36]. Future technology development should prioritize human factors and cognitive ergonomics, ensuring that systems are designed to support parents’ cognitive processes, and future research is needed to evaluate these benefits.

In conclusion, published studies collectively indicate that automated platforms can play a significant role in enhancing discharge communication, leading to better patient outcomes. Ours is the first study that aimed to understand critical engagement in APES-D utilization in the NICU. APES-D User was associated with shorter LOS; however, the tool utilization was strongly influenced by the medical insurance type. Future research is needed to identify all contextual factors and mechanisms influencing participation and effectiveness of digital-engagement interventions to ensure equitable home transition. Novel technology should incorporate cognitive ergonomics to optimize information accessibility, reduce cognitive load, and support decision-making for parents and clinicians at this critical transition point.

## Data Availability

The dataset generated during and/or analyzed during the current study are available from the corresponding author on reasonable request and with the permission of the Indiana University Institutional Review Board.
